# Self-Assembly in the Ferritin Nano-Cage Protein Superfamily

**DOI:** 10.3390/ijms12085406

**Published:** 2011-08-22

**Authors:** Yu Zhang, Brendan P. Orner

**Affiliations:** Division of Chemistry and Biology Chemistry, Nanyang Technological University, 21 Nanyang Link, Singapore 637371, Singapore; E-Mail: zh0010yu@e.ntu.edu.sg

**Keywords:** ferritin, maxi-ferritin, mini-ferritin, self-assembly

## Abstract

Protein self-assembly, through specific, high affinity, and geometrically constraining protein-protein interactions, can control and lead to complex cellular nano-structures. Establishing an understanding of the underlying principles that govern protein self-assembly is not only essential to appreciate the fundamental biological functions of these structures, but could also provide a basis for their enhancement for nano-material applications. The ferritins are a superfamily of well studied proteins that self-assemble into hollow cage-like structures which are ubiquitously found in both prokaryotes and eukaryotes. Structural studies have revealed that many members of the ferritin family can self-assemble into nano-cages of two types. Maxi-ferritins form hollow spheres with octahedral symmetry composed of twenty-four monomers. Mini-ferritins, on the other hand, are tetrahedrally symmetric, hollow assemblies composed of twelve monomers. This review will focus on the structure of members of the ferritin superfamily, the mechanism of ferritin self-assembly and the structure-function relations of these proteins.

## 1. Introduction

Nature uses self-assembly to generate a wide diversity of large, complex, and often highly symmetric protein architectures with a minimum of synthetic remuneration. Establishing the fundamentals of self-assembly is important for achieving an understanding of this important process in general and for its eventual manipulation to generate unique and novel structures.

Most evolved polypeptide chains fold to produce stable three-dimensional structures. This folding of proteins also orientates and projects functional groups on the protein surface that can be recognized and decoded through binding by other proteins. Thus, the information flow defined by the Central Dogma [1] that starts with the genetic code can be expanded through protein folding into protein-protein interactions [2]. These protein-protein interactions are fundamental to defining the self-assembly of large, complex structures.

Self-assembling protein systems form a variety of supramolecular structures with a wide diversity of biological functions or designed properties. These complexes include filaments [3–6], protein lattices [7,8] and symmetric cages [8–11]. Cage architectures have been observed through the self-assembly of viruses capsid [9,10,12], vault [13], heat shock [14–16], DNA binding [17–21] and ferritin proteins [22–24]. These roughly spherical and hollow structures often possess internal icosahedral, octahedral or tetrahedral symmetry which plays an essential role in controlling subunit association. Protein cages have been developed as platforms for nano-structured material synthesis [25], drug delivery [26], cell specific targeting [27] and catalysis [28,29].

The ferritins, a family of protein cages, play a key role in iron sequestration and are highly evolutionary ubiquitous [23,24,30]. The first ferritin was isolated from horse spleen in 1937 [31] and the crystal structure was determined in 1991, which revealed the protein to be a 24-meric cage with octahedral symmetry [32]. Subsequently, ferritins from diverse organisms including animals [33,34], plants [35,36] and bacteria [37–39] have been isolated and crystallized, and these possess structures related to that of horse spleen ferritin. Further studies have demonstrated that the protein cages store excess cellular iron as mineralized hydrous ferric oxide in their cavities. The ability to sequester iron grants ferritins dual functions in iron detoxification and in establishing a cellular iron reserve. Although the DNA and amino acid sequences for the ferritins vary considerably [40] (up to 80%), their well conserved three dimensional tertiary structures indicate identical or similar monomer folding [23]. Their self-assembled cage structures, however, can differ considerably.

The ferritin superfamily can be broken into three sub-families: the classical ferritins (Ftn), the bacterioferritins (Bfr), and the DNA-binding proteins from starved cells (Dps). The Ftn and Bfr proteins are considered maxi-ferritins, whereas Dps proteins are mini-ferritins (see below). These three sub-families share the same characteristic four-helix bundle fold [41,42]. The Ftn proteins are found in all three domains of life (eukarya, archaea and bacteria) and are typical members of ferritin family. The Bfr proteins have identical quaternary structure to the Ftn proteins; however they are restricted to bacteria and archaea. The most significant difference between the Ftn and Bfr proteins is the presence of twelve heme moieties. The Dps proteins form a smaller molecule with a lower iron storage capacity than the Ftn and Bfr proteins and utilize unique ferroxidase sites.

**Figure 1.**
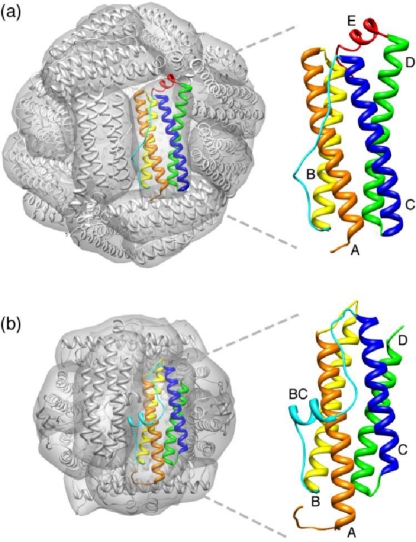
Typical structures of octahedral maxi-ferritin (**a**, PDB ID: 1 bfr) and tetrahedral mini-ferritin (**b**, PDB ID: 1 dps). Their four-helix bundle monomers are shown as ribbons. The A helix is colored orange; the B helix, yellow; the BC loop in the maxi-ferritin or BC in the mini-ferritin helix, cyan; the C helix, blue; the D helix, green and the E helix, red.

**Figure 2.**
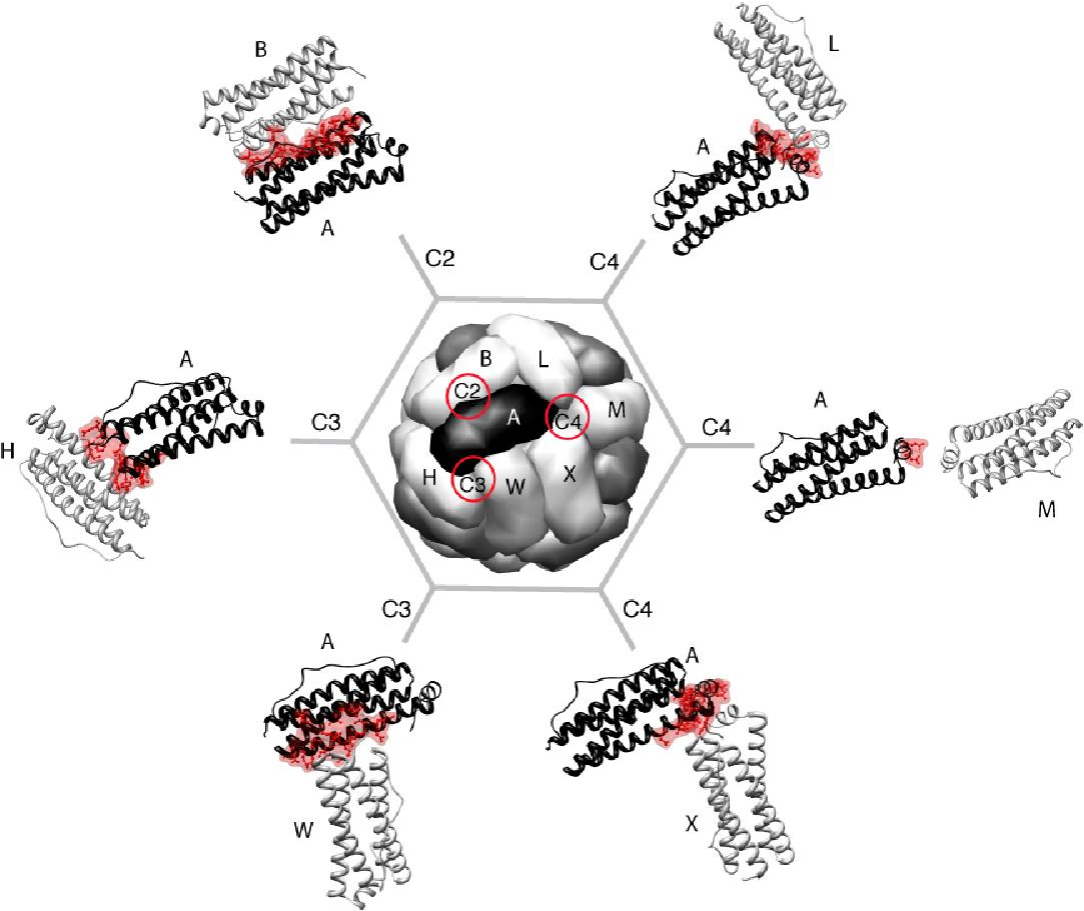
Each monomer (for example monomer A) in *E. coli* bacterioferritin, a maxi-ferritin, is involved in six unique inter-subunit interactions at the respective symmetry related interfaces (C2, C3, C4 are highlighted with red circles). The subunits (B, H, W, X, M and L) which interact with subunit A are indicted. The residues in subunit A that are involved in the inter-subunit interactions are shown in red. The figure is generated using UCSF Chimera [44] (PDB ID: 1 bfr).

Maxi-ferritins are composed of twenty-four identical or homologous subunits (∼20 kDa) that assemble into a large spherical cage (outer diameter ∼120 Å) with a hollow cavity (inner diameter ∼80 Å). Mammalian ferritins often consist of two types of similar subunits, heavy (H) and light (L) chain, with a molecular weight of ∼21 and ∼19 kDa respectively. The cavity can accommodate up to ∼4500 Fe atoms in the form of a hydrous ferric oxide mineral core with variable amount of phosphate [43]. Each monomer is made up of a four-helix bundle (the A, B, C and D helices) with a short fifth helix (the E helix) at the *C*-terminus (Figure 1(a)). In the octahedral cage structure (432 point group symmetry), each subunit interacts with six adjacent monomers through three types of symmetry-related interfaces. There are twelve dimerization interaction interfaces at the two-fold axes, eight trimerization interaction interfaces at the three-fold axes and six tetramerization interfaces at the four-fold axes (Figure 2).

**Figure 3.**
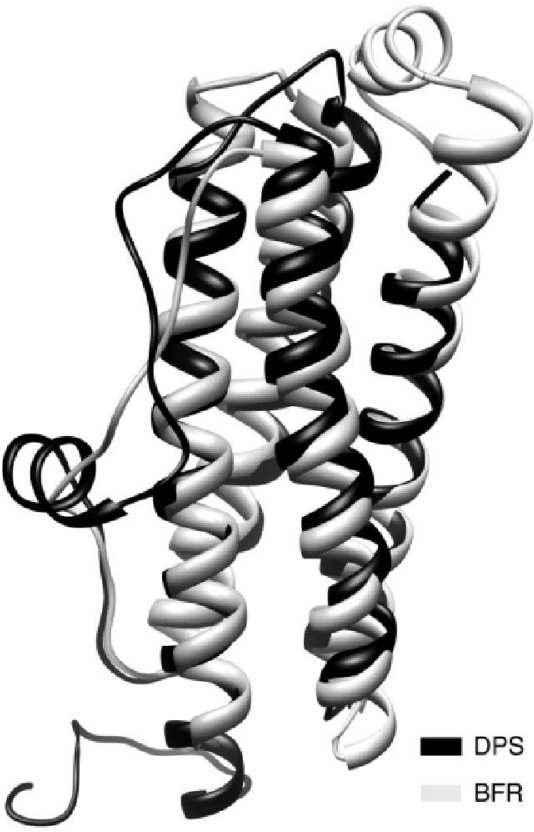
Superimposition of *E. coli* bacterioferritin (grey) and *E. coli* DPS (dark) monomers generated using the DaliLite online program [50,51]. The RMSD of the structural superimposition is 2.1 Å (PDB ID: 1 bfr, 1 dps).

The mini-ferritin Dps proteins form cage-like oligomers similar to the maxi-ferritins but made up of only twelve monomers. The first Dps protein family member was isolated from *E. coli* grown in starvation conditions in 1992. Along with protecting bacteria from oxidative damage, it also forms an extremely stable complex with DNA without apparent sequence specificity [45]. The crystal structure of dodecameric *E. coli* Dps reveals a hollow protein with 32 (tetrahedral) point group symmetry demonstrating that Dps is a structural analogue of the maxi-ferritins [17], although the main function of Dps proteins is iron detoxification as opposed to iron storage. The Dps dodecamer measures ∼9 nm in diameter and has a central cavity of ∼ 4.5 nm which can hold an iron core of up to ∼500 Fe^3+^ iron ions [17]. Similarly to the maxi-ferritins, the Dps monomer folds into a four-helix bundle (the A, B, C and D helices). However, unlike the maxi-ferritin, the loop between the B and C helices forms a short helix (Figure 1 and Figure 3). The BC helix runs nearly orthogonal to the four-helix bundle axis and is exposed on the outside of the assembled protein cage. Moreover, the Dps monomer contains no E helix as is found in the maxi-ferritins.

**Figure 4.**
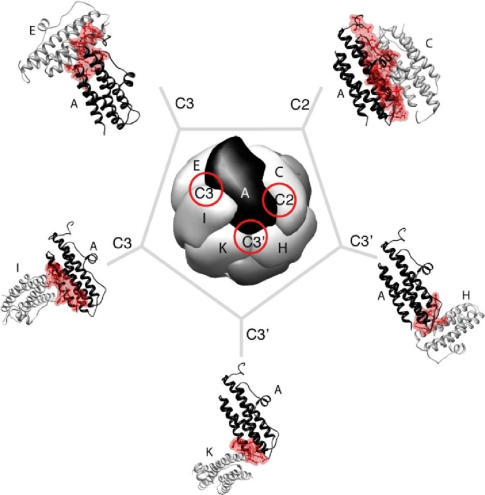
Each monomer (for example monomer A) in *E. coli* DPS, a mini-ferritin, is involved in five unique inter-subunit interactions at the respective symmetry related interfaces (C2: two-fold; C3: ferritin-like three-fold; C3′: Dps-like three-fold axes are highlighted with red circles). The subunits (B, H, W, X, M and L) which interact with subunit A are indicted. The residues in subunit A that are involved in the inter-subunit interactions are shown in red. The figure is generated using UCSF Chimera [44] (PDB ID: 1 dps).

**Figure 5.**
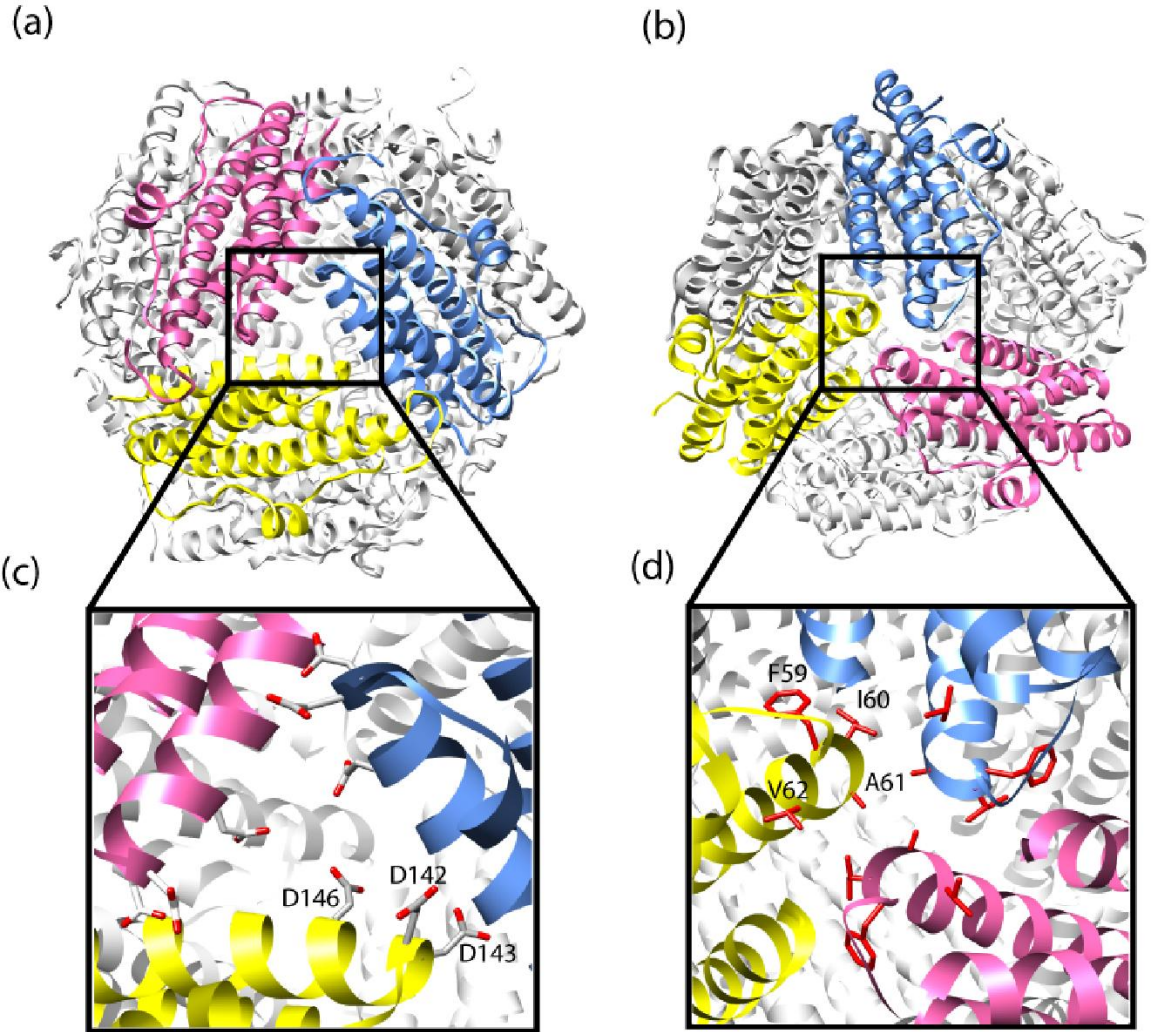
DPS mini-ferritin protein cages viewed along the (**a**) ferritin-like three-fold axis; and (**b**) Dps-like three-fold axis; (**c**) The expansion shows the aspartic acid residues lining the ferritin-like pore; (**d**) The expansion shows the hydrophobic amino acids lining the DPS-like pore. Note: Due to symmetry, the residues in (c) and (d) are labeled on only one monomer. The figure is generated using UCSF Chimera [44] (PDB ID: 1 dps).

In mini-ferritins, each subunit interacts with five surrounding monomers through two types of symmetry-related protein-protein interfaces. Six dimer interactions are at two-fold symmetry axes, and four trimerization interactions are centered at the three-fold axes (Figure 4). There are two major differences in the protein packing which forms the supra-structures of the mini-ferritins and the maxi-ferritins. First, due to the absence of the E helix, which is crucial for defining the four-fold symmetry and hence the tetrameric interactions in maxi-ferritins, mini-ferritins do not form an analogous interface. Thus Dps forms a smaller, lower symmetry oligomer. Secondly, the maxi-ferritins’ octahedral, symmetry determines that only one type of trimeric interaction is formed as the chemical environment around the three-fold axes is identical on the “front” and “back” sides of the assembled cage [17]. In contrast, two types of nonequivalent three-fold interfaces exist in the mini-ferritin tetrahedral dodecamer [17]. One type of symmetric trimer interface is formed by the *N*-terminal ends of the monomers and is called “ferritin-like” as the packing is similar to that in a maxi-ferritin (Figure 5 (a)). The second type is formed by the *C*-terminus of the monomers. This interface is unique to this protein family; hence it is called “Dps-like” (Figure 5 (b)).

Both the maxi- and mini-ferritins utilize the electrostatic potential generated by the negatively charged residues lining the pores at the *N*-terminal, ferritin-like interface to help iron enter and exit the protein inner cavity [46,47] (Figure 5 (c)). The pore formed at the unique *C*-terminal, Dps-like interface of the mini-ferritins is smaller, due to hydrophobic constriction, and is less acidic [17] (Figure 5 (d)). Therefore, it most likely plays no role in iron transport.

Another significant difference between mini- and maxi-ferritins is the position of the ferroxidase sites. In the maxi-ferritins it is located in the middle of the monomeric four-helix bundle whereas, in Dps, it is situated at the interface between two-fold axis-related monomers [48]. Moreover, the ability of Dps proteins to bind non-specifically to DNA through *N*- or *C*-terminal extensions is another feature that distinguishes Dps proteins from maxi-ferritins [17,45,49].

Although the maxi- and mini-ferritin proteins fold into remarkably similar monomer structures, their self-assembled architectures are distinct. They both form cages however they have different symmetries and oligomerization states. Understanding the fundamentals of their self-assembly may shed light on the nature of these unique structures. As protein-protein interactions define the interfaces that are responsible for stitching together the monomers, they may be key in establishing a deep enough appreciation of these highly symmetrical constructions to be able to manipulate them into structures with novel functions or unique properties. This review will focus on the proposed rules governing and pathways and mechanisms controlling the self-assembly of maxi- and mini-ferritins with an emphasis on protein-protein interactions.

## 2. Maxi-Ferritin Self-Assembly

### 2.1. Proposed Pathways of Maxi-Ferritin Assembly

Resolving the self-assembly mechanism of protein supramolecular complexes is difficult especially if the structures are highly symmetrical and homo-oligomeric. Moreover, the characterization of the various thermodynamically and kinetically accessible intermediates for the association and disassembly pathways can help achieve mechanistic insight, however, these studies can be challenging due to difficulties with the determination of folding and assembly rate-limiting steps and coupling between the two [52]. Thus, only a few studies have explored the self-assembly mechanism of maxi-ferritins.

X-ray diffraction analysis into the assembly mechanism of horse spleen apoferritin suggests that stable dimers act as assembly intermediates [53]. Furthermore, sedimentation velocity analytical ultracentrifugation of this protein at various pHs demonstrates the stability of dimers in solution [54] and also suggests that assembly proceeds from dimers to tetramers and octamers. The overall assembly mechanism of horse spleen apoferritn was first proposed by Gerl *et al*. [55] who interpreted data obtained through intrinsic fluorescence, far-UV circular dichroism and glutaraldehyde cross-linking experiments. It was observed that the completely self-assembled product was formed through a series of concentration dependent association reactions involving a mixture of partially assembled subunits. These subunits include the “structured monomer”, dimer, which was the most highly populated species, trimers, hexamers, in small amounts, and dodecamers. The overall proposed mechanism is described by the following scheme:

$$24M_{1}^{*}\to 24M_{1}\;\to \;8M_{1}+8M_{2}\;\to \;8M_{3}\;\to \;(4M_{6})\to \;2M_{12}\;\to \;M_{24}$$

where M^*^_1_ is the unfolded monomer, M*_i_* are intermediates with i folded monomers and M_24_ is the completely self-assembled ferritin. In this model, to initiate assembly, the unfolded monomers must first acquire a native-like conformation to generate “structured monomers” which provide complementary interfaces and subsequently dimerize at high rate. This mechanism was supported by further crosslinking experiments [56] where dimers, trimers and tetramers were isolated to investigate their capacity for reassociation. It was shown that two hexamers could be used to form a dodecamer, and two dodecamers could assemble into a 24-mer. These results led to a refined model where the 24-meric cage assembles from dimer (M_2_) via tetramers (M_4_) and hexamers (M_6_). This mechanism is supported by Banyard *et al*. [53], who proposed that the monomer is expected to be unstable, and the stable tetramer and hexamer intermediates can be thought of as dimers of dimers and trimers of dimers respectively. However, the cross-linking experiments suggested that both dimer and monomer are involved in the formation of a stable trimer, hexamer is a transient intermediate that could only be detected in small amounts and tetrameric and octameric intermediates are undetectable. While these results don’t support the mechanism, they don’t completely rule it out in that it is possible to imagine a model where some of the isolatable oligomerization states are unproductive assembly dead-ends that need to completely or partially disassemble before forming a productive, albeit short lived, intermediate.

Horse spleen apoferritin is highly resistant to chemical denaturation, pH changes and heat [57,58]. Recently, though, it was shown through quantitative data analysis of SAXS measurements that apoferritin can undergo stepwise disassembly through several structural intermediates below pH 3.40 [59]. The dissociation process starts with hollow spherical structures with two holes, followed by “headphone”-shaped structures, and ultimately, rod-like oligomers (mainly trimers) or monomers. The structural recovery of the intermediates during the pH-induced reassembly process is dependent on the history of the disassembly process; for example the hollow sphere with the double hole defect could never be recovered back to the intact hollow sphere. How this data relates to proposed mechanisms of assembly has not been fully explored.

### 2.2. Mutation Related Studies on the Effect of Maxi-Ferritins Self-Assembly

Various mutations and modifications of maxi-ferritins have been studied to evaluate the role that various residues and regions play in protein assembly and function. Through this process, many mutations have been discovered that have little to no effect on the self-assembly of the cage, demonstrating their robustness, which bodes well for the utility of these proteins in engineering and bio-conjugation applications. Conversely, mutations have been discovered that radically affect the self-assembly though they are relatively conservative and in some cases are even single point mutations. These cases dramatically emphasize the possibility of rationally manipulating the properties of these proteins with precision.

One early study involved engineering of *E. coli* BFR to replace the *C*-terminal residues (REEG) with the eighteen residue λ peptide (RLPFTSCAVCLQDSMRSR) [22]. The *C*-terminus in this maxi-ferritin is positioned at the end of the E helix and thus points inside the cavity suggesting the peptide extension would also be present inside the assembled ferritin. Gel filtration demonstrated that the mutant exists solely as 24-mer, whereas the wild type is a mixture of 24-mer and dimer. The greater assembly stability of the variant with respect to wild type BFR implies that the *C*-terminal extensions at the C4 interface (the interactions between monomers A and L and A and M in Figure 2) are forming additional contacts. However, crystallization and *in vitro* iron uptake experiments suggest that the peptide extension is possibly blocking access of iron to the central cavity.

The E helix from the *C*-terminus of *E. coli* BFR was found to exhibit great power in controlling the assembly. Removal of the E helix from BFR resulted in a destabilized protein that could only assemble into a dimer [60]. However, Luzzago and coworkers [61] reported that the E helices along the four-fold symmetry axis are not essential for human ferritin H-chain assembly and proposed that the ferritin can assemble into a cage with the E helix either flipped out or flipped inside the central cavity. These conflicting results suggest that the role the E helix plays in ferritin self-assembly may be protein specific.

Arosio and coworkers [62] deleted twenty-two residues at the *C*-terminus in human H-chain ferritin including the E helix along with an unstructured *C*-terminal tail. This mutant assembled and maintained the ability to catalyze iron oxidation. However, if the mutation involved six additional *C*-terminal residues to include a total of twenty-eight amino acids, the mutant failed to assemble [63] indicating that six amino acids at the end of the *C*-terminal tip of the D helix are essential for human H-chain ferritin self-assembly. The unstructured *C*-terminal tail of human ferritin was further explored by Ingrassia *et al*. [64] who found that modification of the last six-residues had no major effect on the physical properties of ferritin, however the solubility and assembly decreased progressively with the extension of the tail suggesting that this part of the protein plays some, although minor, role in assembly.

Modifications at the *N*-terminus and the loop between the B and C helices of maxi-ferritins have also been investigated to examine the role that these regions play in protein assembly. Arosio and coworkers [62] engineered human H-chain ferritin by deleting the first thirteen residues at the *N*-terminus and found that the protein could still assemble and also catalyze iron oxidation. This is not particularly surprising as the *N*-terminus points outside of the protein cage and has little protein-protein overlap in most family members. Yohizawa *et al*. [65] deleted four and eight residues at the *N*-terminus of horse L-chain apoferrtin and found that one mutant only formed dimers at or below pH 2.0 leading to the hypothesis that polar interactions at the *N*-terminus are responsible for the decreased stability in acidic conditions. Another study investigated the role of the BC loop by deleting two residues (L82 and I85) from human H-chain ferritin. These simple mutations resulted in disassembly of the cage probably resulting from disruption of the dimer assembly intermediate (see above).

Further modification of bacterial proteins were performed by Fan *et al*. [60], where several mutants were constructed by swapping the E helix from *E. coli* BFR and the BC helix from DPS to determine how these two structural elements affect the stability and oligomerization of the two proteins with respect to each other. The E helix from the *C*-terminus of BFR was found to exhibit great control in the assembly. Fusion of the BFR E helix to DPS resulted in a protein that discretely assembles into a 12-mer with a size uniquely intermediate between the two parents. The BC helix from DPS, on the other hand, plays less of a role in stabilizing oligomerization. Removal of the BC helix from DPS displayed insignificant changes in the oligomerization state and BFR remained as a mixture of dimer and 24-mer despite addition of the BC helix.

Mutation-based studies can help to understand the role that certain residues, and hence specific interactions play in ferritin protein self-assembly, or its underlying folding, in addition to identifying potentially relevant assembly intermediates. Santambrogio *et al*. [66] reported that the renaturation of human H-chain ferritins mutated around the four-fold, three-fold and two-fold axes (L169R at the four-fold axis, D131I/E134F at the three-fold axis, I85C at the two-fold axis) yielded assembly intermediates ranging from monomers and dimers to all types of higher oligomers. Any of these intermediates could be induced to assemble into 24-mer either through concentration or by co-renaturing them with wild-type H-ferritin. However, as all pathways required initial assembly of dimers, these were proposed to be the essential intermediates for assembly.

Kilic *et al*. [67] modified the bacterioferritin from *R. capsulatus* by site-directed mutagenesis to result in proteins that assembled into discrete dimers. The amino acids Glu128 and Glu135, located at an interface similar to that described in Figure 2 (between monomers A and H), were mutated to either alanine or arginine to determine their role in stabilizing the 24-mer. It was found that E128A/E135A and E128R/E135R double mutants formed stable dimers, strongly suggesting that the interactions involving Glu128 and Glu135 contribute significantly to the stabilization of the assembled 24-meric cage. The X-ray structures of *E. coli* and *R. capsulatus* BFR [38,68] reveal that the conserved Glu128 and Glu135 on one monomer are most likely interacting with Arg61 and the *N*-terminal amine of another monomer. The importance of salt bridge formation between Glu128 and Arg61, and its significance in self assembly of the cage in *E. coli* bacterioferritin was also reported by Zhang *et al*. [69]. It was observed that E128A and R61A single mutants formed stable dimers in solution. Interestingly, R61A possessed higher thermostability than the wild type bacterioferritin. This report also disclosed that two other key amino acids; Y114 at the three-fold axis and R30 located at the two-fold axis also play significant roles in self-assembly.

The importance of water pockets at the protein-protein interfaces defined by the two-fold symmetry axes was demonstrated by Ardejani *et al*. [59]. Assisted by computational analysis, it was proposed that stabilizing point mutations could be achieved by bridging the water pocket associated with N23. All three predicted stabilizing mutations, N23L, N23F and N23W, assembled into cages and possessed a higher thermal stability than wild type BFR. One of the mutants, N23F, appeared to push the oligomerization state toward the 24-mer at the expense of the dimer, whereas the remaining two mutants had an opposite effect. These results, taken together, suggested that mutation at the two-fold interface could possibly result in a more stable but “unproductive” dimer which forms a geometry incompatible with cage formation. This proposal complicates the mechanism of assembly, but potentially explains previously unresolved experimental data (see above).

## 3. Mini-Ferritin Self-Assembly

### 3.1. Proposed Pathways of Mini-Ferritin Assembly

Compared to that of the maxi-ferritins, the self-assembly of dodecameric mini-ferritins has not been as widely explored. One of the most extensively studied min-ferritins, is the *Mycobacterium smegmatis* Dps protein. Vijayan and coworkers proposed that the protein cage can be assembled in three different ways. First, the monomers could form two-fold symmetric dimers, which would later assemble into a dodecamer with 32 symmetry. The other two self-assembly mechanisms include forming either of the two types of three-fold symmetric trimers followed by assembly into the tetrahedral dodecamer [70].

Supporting a trimer-dependent assembly mechanism, the presence of these intermediates was experimentally observed. Two stable oligomeric forms of *M. smegmatis* Dps, a trimer and a dodecamer were detected by Gupta *et al*. [71]. The conversion between the trimeric and dodecameric could be achieved by incubation at 37 °C for 12 h. Interestingly, it was found that these two oligomeric forms have different DNA binding affinities which may be due to a simple multivalent effect [72].

### 3.2. Mutation Related Studies on the Effect of Mini-Ferritins Self-Assembly

The role of the *N*- and *C*-terminus of *M. smegmatis* Dps in the self-assembly of the dodecameric protein cage was examined by Vijayan and coworkers [73]. A protein with sixteen *C*-terminal residues deleted could assemble whereas if twenty-six residues of the *C*-terminus were deleted, no cage was observed, emphasizing the importance of the *C*-terminus in self-assembly. Deletion of the short *N*-terminal tail resulted in a protein that formed trimer instead of dodecamer in solution but could fully assemble when crystallized, emphasizing that this region also is important. Recalling that the *N*-terminus and *C*-terminus of DPS lies at the ferritin-like and Dps-like three-fold symmetry axes, respectively, and that the trimer has been implicated as a DPS folding intermediate (see above), these results can shed light on the assembly mechanism when taken together. The fact that disruption of both these interfaces results in proteins with impaired cage formation implies a near-equal role for these two types of trimers in a mechanism of self-assembly.

The same research group identified clusters of amino acids on various interfaces of *M.s.* Dps and predicted key residues that could disrupt the assembly upon mutation [74,75]. Two mutations, E146A at the ferritin-like trimer interface and F47E at the Dps-like trimer interface, were constructed in an attempt to reinforce the predications. Despite predicting that E146A would impair trimer formation, the mutant could form either trimer or docamer at different temperatures. On the other hand, F47 was found to be crucial for dodecamerization as the single mutant F47E and double mutant E146AF47E were observed as trimer and monomer respectively in solution with no observed cage. These results, although with some caveats, suggest that the ferritin-like trimer may play a more important role in the assembly mechanism.

Unlike maxi-ferritins, the loop between the B and C helices in mini-ferritins forms a short helix. Fan *et al*. [60] reported that the deletion of BC helix in *E. coli* Dps results in a mutant that can still form a cage, indicating that the BC helix is not essential to self-assembly.

### 3.3. pH Effects on the Self-Assembly of Mini-Ferritin

Changing the pH can help to identify key electrostatic forces controlling the protein structure as changes in proton concentration can change the net protein charge. In oligomeric proteins, the protonation and deprotonation of amino acid residues as a result of pH change can give rise to repulsive interactions that lead to loss of quaternary, tertiary and even secondary structure. Therefore, protein structural changes induced by changes in pH may lend insights into the electrostatic forces involved in subunit recognition and association, and also on the possible mechanisms for the assembly and disassembly processes.

The stability of *L. innocua* Dps protein in acidic conditions was investigated by Chiaraluce *et al.* [76] and the protein was found to be extremely pH stable. Dissociation into dimers was observed below pH 2.0 and further dissociation to monomers with significant secondary structure loss was observed at pH 1.0 [76]. The study suggested that the basic structural unit is dimer which, because it is resistant to pH, is held together mainly by hydrophobic interactions.

Examination of the pH stability of *M. smegmatis* Dps by Ceci *et al*. [21] revealed that *M.s.* Dps dissociated reversibly into dimers at conditions above pH 7.5 and below 6.0. Furthermore, dimers dissociate to monomers at pH 4.0. The two dissociation steps were attributed to the destruction of salt bridges between Glu157 and Arg99 and Asp66 and Lys36 located at the three fold symmetry axes and across the dimer interface respectively.

## 4. Conclusions

Ferritin proteins self-assemble into multi-subunit, hollow, nano-scale cages. They have been the focus of much recent attention as part of bioorthogonal methodology development, drug delivery studies, and as platforms of nano-structured materials. Further investigations into the self-assembly mechanism of the ferritin superfamily proteins not only shed light on the fundamentals of protein folding and protein-protein interactions, but also assisted in rationalizing protein engineering to form more stable nanostructures, with the ultimate goal of utilizing them as vehicles for delivery systems and in novel structure-based materials.
